# Biomolecular characterization of 3500-year-old ancient Egyptian mummification balms from the Valley of the Kings

**DOI:** 10.1038/s41598-023-39393-y

**Published:** 2023-08-31

**Authors:** B. Huber, S. Hammann, C. E. Loeben, D. K. Jha, D. G. Vassão, T. Larsen, R. N. Spengler, D. Q. Fuller, P. Roberts, T. Devièse, N. Boivin

**Affiliations:** 1 Department of Archaeology, Max Planck Institute of Geoanthropology, Jena, Germany; 2 Institute for Archaeological Sciences, Eberhard Karl University of Tübingen, Tübingen, Germany; 3 Department of Chemistry and Pharmacy, Friedrich-Alexander Universität Erlangen-Nürnberg, Erlangen, Germany; 4 Egyptian and Islamic Collections, Museum August Kestner, Hannover, Germany; 5 Department of Biochemistry, Max Planck Institute for Chemical Ecology, Jena, Germany; 6 Domestication and Anthropogenic Research Group, Max Planck Institute of Geoanthropology, Jena, Germany; 7 Institute of Archaeology, University College London, London, UK; 8 isoTROPIC Research Group, Max Planck Institute of Geoanthropology, Jena, Germany; 9 Centre Européen de Recherche et d’Enseignement des Géosciences de l’Environnement (CEREGE), Aix Marseille University, Aix-en-Provence, France; 10 School of Social Science, The University of Queensland, Brisbane, QLD Australia

**Keywords:** Biochemistry, Biogeochemistry, Environmental social sciences

## Abstract

Ancient Egyptian mummification was practiced for nearly 4000 years as a key feature of some of the most complex mortuary practices documented in the archaeological record. Embalming, the preservation of the body and organs of the deceased for the afterlife, was a central component of the Egyptian mummification process. Here, we combine GC–MS, HT-GC–MS, and LC–MS/MS analyses to examine mummification balms excavated more than a century ago by Howard Carter from Tomb KV42 in the Valley of the Kings. Balm residues were scraped from now empty canopic jars that once contained the mummified organs of the noble lady Senetnay, dating to the 18th dynasty, ca. 1450 BCE. Our analysis revealed balms consisting of beeswax, plant oil, fats, bitumen, Pinaceae resins, a balsamic substance, and dammar or *Pistacia* tree resin. These are the richest, most complex balms yet identified for this early time period and they shed light on balm ingredients for which there is limited information in Egyptian textual sources. They highlight both the exceptional status of Senetnay and the myriad trade connections of the Egyptians in the 2nd millennium BCE. They further illustrate the excellent preservation possible even for organic remains long removed from their original archaeological context.

## Introduction

Ancient Egyptian society is renowned, in academic and public circles alike, for the complex rituals and extraordinary material culture that it attached to death, particularly amongst ruling social elites^1^. Already by the Late Neolithic, funerary monuments had emerged as central points on the landscape for agricultural groups inhabiting the Nile floodplain^2^. Later, monumental structures, from the earliest built mastabas ca. 3000 BCE to the renowned pyramids of Giza ca. 2600 BCE^3^, rose to become key elements of Egyptian religion, economy, society and politics^4^. So important was the elaboration of the funerary sphere in ancient Egyptian culture that its necropolises have been characterized as ‘cities of the dead’^2^.

At the epicenter of this rich funerary culture were the buried individuals themselves, who were subjected to a highly complex set of postmortem mummification processes that, with the exception of some examples in Chile and China^5–7^, are unparalleled in the archaeological record. Ancient Egyptian mummification predates the First Dynasty, as evident in embalming remains found in Late Neolithic burials^8^, and continued all the way through to the Greco-Roman period^9^, making it a core feature of Egyptian funerary archaeology. Contrasting with natural mummification, which can occur under arid conditions like those found in the Egyptian desert, artificial mummification in Egypt entailed evisceration, and the deliberate desiccation and preservation of the body through the application of various substances^10,11^. The mummification procedure encompassed the meticulous removal of organs such as the lungs, liver, stomach, and intestines, followed by embalming^12^. The organs were frequently, but not always, mummified and stored in separate canopic jars. This practice served the purpose of facilitating corporal desiccation by inhibiting bacterial and fungal growth. Its objective was to ensure the long-term preservation of the deceased's body for the afterlife, providing a vessel for the return of the individual's 'souls', in line with Egyptian belief systems^11,13^. The ancient Egyptians held a multifaceted view of the 'soul', conceiving it as a composite of several elements, most notably the *Ka*, *Ba and Akh,* which were associated with notions of the afterlife and funerary rituals^14,15^.

**Figure 1:**
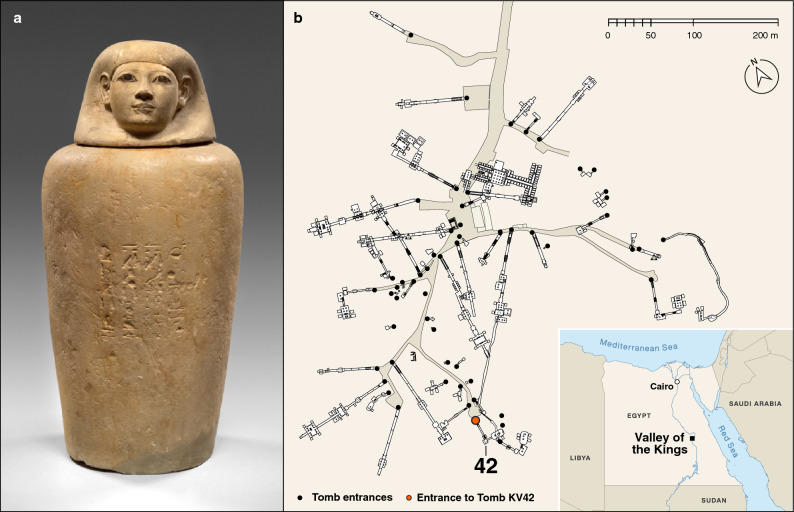
(**a**) Canopic jar of Senetnay, “Wet Nurse of the King” (Amenhotep II), which originally contained Senetnay’s mummified lungs, as evident from the inscriptions on the vessel referring to Nephthys, the protective goddess of the lungs. Height of the jar with lid: 42.4 cm; height without lid: 33.7 cm; max. diameter: 21.5 cm. © Museum August Kestner, Hannover (Germany); photo: Christian Tepper (museum’s photographer). (**b**) Map of the Valley of the Kings with the location of Tomb KV 42, where the canopic jars were found. Sources of maps: Weeks, Kent R. (ed.). Atlas of the Valley of the Kings (= Publications of the Theban Mapping Project, 1). Cairo: American University in Cairo Press, 2000, 2003. Available online at https://thebanmappingproject.com/sites/default/files/plans/Valley%20of%20the%20Kings.pdf, and Natural Earth vector map data (maps were created using QGIS 3.12 (https://qgis.org/en/site/)).

Examples of mummified organs were discovered by Howard Carter in the royal tomb “KV (Kings’ Valley) 42” in Thebes (now Luxor) in 1900^16^. The viscera he encountered in Tomb KV 42 belonged to the noble lady Senetnay, who lived in Egypt around 1450 BCE. She was the wet nurse of the long-awaited son and heir of Pharaoh Thutmose III, the future Pharaoh Amenhotep II, who was nurtured and breastfed by Senetnay during infancy^17^. After her death, Senetnay’s mummified organs were carefully stored in four canopic jars with lids in the shape of human heads (Fig. 1). In order to preserve her remains for the afterlife, they were embalmed, ensuring their long-term conservation, ostensibly for eternity. Two of the jars, those made to contain Senetnay’s lungs and liver, are now held in the Egyptian collection of the Museum August Kestner, Hannover (Germany)^17,18^. While the mummified organs themselves have been lost, and the jars are presently empty, residues of the mummification balms are partially preserved as thin coatings on the walls and bases of the jars, as well as permeating into the porous limestone of which the jars are made.

The exact recipes used in ancient Egyptian mummification balms have long been debated due to the paucity of ancient Egyptian texts naming their precise ingredients^19^. Despite the long period over which mummification was practiced (almost 4000 years), there are only a few written sources—such as the *Ritual of Embalming*^20^—that address the mummification process, and none of these texts provide the exact ingredients used in the preparation of the balms. Historical descriptions from much later Greek and Roman sources (e.g., Herodotus, Diodorus Siculus^21,22^) do specify some ingredients, but these were not necessarily the same as those employed more than a millennium earlier. For these reasons, the use of molecular analyses to help in identifying the ingredients of ancient Egyptian embalming materials has been of great interest to scientists since the late 1970s^23^. In particular, technological advances in gas chromatography and mass spectrometry techniques have contributed significantly to elucidating the chemistry of ancient Egyptian balms^24,25^. Previous studies have identified a number of different ingredients that were used in the production of mummification balms, in various configurations, such as oils and fats^9,26–31^, beeswax^9,29,30,32,33^, bitumen^8,28,34,35^, gums and sugars^8,9,32^, and resins and tars^8,27,28,31,33,34,36–41^. However, most of these studies focused on embalming materials obtained from the bandages and tissues of mummies themselves, and only a few studies have been carried out on the substances used to embalm the accompanying organs in canopic jars^28,33,42^ (see also the Canopic Jar Project at the University of Zurich).

Here, in order to elucidate what broader social, technological and cultural insights can be acquired from the balms used to mummify organs, we investigate balm samples from two of the canopic jars belonging to Senetnay (the other two jars belonging to the assemblage are not available for analysis, as one is housed at the Egyptian Museum in Cairo while the location of the other remains undetermined). The jars analyzed are those that contained Senetnay’s lungs and liver, and were initially stored in Egypt, then in the private collection of the Egyptologist Friedrich Wilhelm Baron von Bissing in Munich, then subsequently at the Museum Carnegielaan 12 in The Hague, and finally, since 1935, in the Egyptian collection of the Museum August Kestner in Hannover (with two years of security storage in a salt mine in Grasleben during World War II)^17,18^. At each location, over more than 123 years, the remains were stored under more or less ideal “museum conditions”. Understanding the complexity of ancient organic residues, especially mixtures of different products, requires the analysis of multiple compound groups. In recognition of the chemical diversity of the biological components in many analyzed mummification balms, we draw upon a multi-analytical approach combining gas chromatography mass spectrometry (GC–MS), high temperature gas chromatography mass spectrometry (HT-GC–MS) and liquid chromatography tandem mass spectrometry (LC–MS/MS) to differentiate and identify the organic substances contained within Senetnay’s canopic jars.

## Results

A total of 6 balm samples were selected for analysis, comprising one sample from the bottom and two samples from the inner walls of each of the canopic jars (see Supplementary Figure S1 and Table S1 for exact location and description of samples). The balm samples were subjected to a series of extraction/dissolution steps followed by LC–MS/MS, GC–MS and HT-GC–MS analyses. The results, detailed below, show good preservation of molecules in the samples taken from the interior bottoms of the jars (i.e., AES 062 from jar 1 and AES 067 from jar 2), where residual layers of the embalming material remained adhered. In contrast, residues scraped from the inner walls, which were partially absorbed into the limestone of the jar and barely visible to the naked eye, demonstrated poorer molecular preservation.

### LC–MS/MS screening for biomarkers of plant exudates and resins

Three compound groups were identified in the extracts analyzed by LC–MS/MS in multiple reaction monitoring (MRM) mode: terpenoids, phenols and aromatic compounds (Table 1). The LC–MS/MS results showed a high abundance of di- and triterpenoids in the embalming material. Predominant among the diterpenoids is 7-oxo-dehydroabietic acid (7ODHA, Table 1), which was observed in all samples. 7ODHA is an oxidized derivative of the diterpene dehydroabietic acid (DHA), which was also present to a lesser extent. Both compounds are characteristic of coniferous plant products, specifically resins from the Pinaceae family, including pine (*Pinus* spp.), larch (*Larix* spp.) and cedar (*Cedrus* spp.)^8,9,28,34,41^. Other compounds characteristic of Pinaceae resins were also included as analytical standards for the optimization of MRM parameters, notably pimaric, isopimaric, palustric and neoabietic acids^43^. However, due to similar retention times, fragmentation patterns and molecular weights (302 g/mol), LC–MS/MS and detection in MRM mode were not sufficient to differentiate them. Therefore, pimaric acid, isopimaric acid, palustric acid and neoabietic acid are summarized in Table 1 as ‘resin acids’ (see also Supplementary Table S5), and the samples were additionally analyzed by GC–MS in order to differentiate these compounds (see GC–MS and HT-GC–MS analysis).

**Table 1:** LC–MS/MS findings from archaeological samples AES 062, 064 and 066 from canopic jar 1 (containing the lungs) and AES 067, 068 and 069 from canopic jar 2 (containing the liver). Constituents identified based on less than 10,000 counts are indicated as ‘trace’. See also Supplementary Table S7 for identified compounds.

Compounds	Canopic jar 1			Canopic jar 2		
	AES 062	AES 064	AES 066	AES 067	AES 068	AES 069
	Interior, bottom	Inner wall	Inner wall	Interior, bottom	Inner wall	Inner wall
Resin acids (pimaric, isopimaric, palustric and neoabietic acids)	✓	Trace	✓	✓	✓	Trace
7-Oxodehydroabietic acid	✓	✓	✓	✓	✓	✓
Dehydroabietic acid	✓	×	✓	✓	✓	✓
Dammarenolic acid	✓	×	×	×	×	×
Oleanonic/moronic acids	✓	×	×	×	×	×
Benzoic acid	✓	✓	✓	✓	✓	✓
Vanillic acid	✓	Trace	Trace	✓	Trace	Trace
Coumarin	✓	Trace	×	✓	×	×

**Figure 2:**
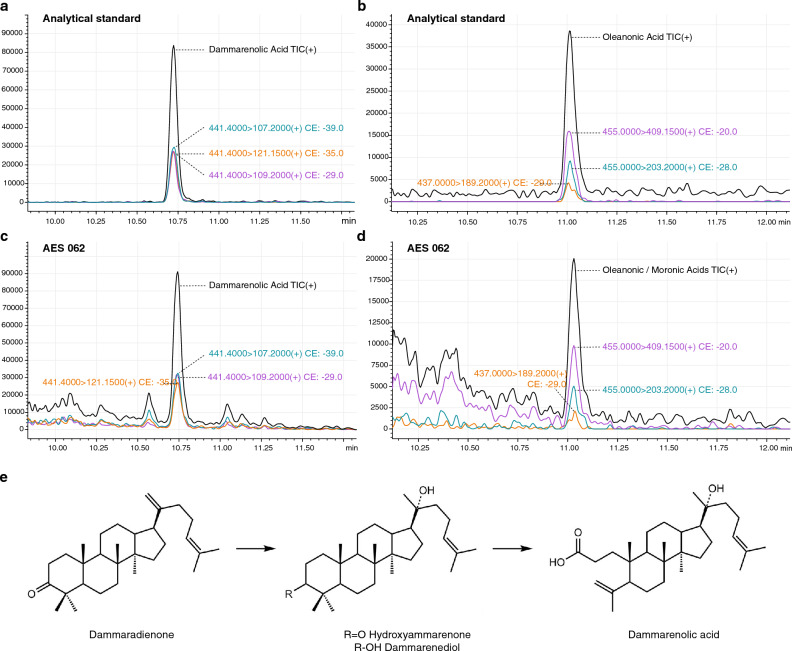
Multiple reaction monitoring (MRM) HPLC chromatograms of the analytical standards dammarenolic acid (**a**) and oleanonic acid (**b**) compared to the presence of these compounds in sample AES 062 of canopic jar 1 (**c,d**). (**e**) Chemical structures of sequential oxidation stages of dammarane-type molecules ^after47^.

The triterpenoids detected in the samples suggest the use of additional scented resinous substances. Dammarenolic acid (Fig. 2a, c), the main secondary metabolite of dammar resin^44^, was present in sample AES 062 of canopic jar 1. This triterpenoid compound is a dammarane-type molecule, but with the opening of the A-ring due to oxidation and breakage of the C–C bond, resulting in a carboxyl functional group^45^. Interestingly, this compound was only detected in jar 1 and not in any of the samples taken from canopic jar 2. Our analysis also revealed a peak corresponding to either oleanonic or moronic acid, two pentacyclic triterpenoids that have similar structures and ionization behaviors, and are accordingly difficult to distinguish. This peak was, however, only detected in low abundance (Fig. 2b, d). Oleanonic acid and moronic acid are typical biomarkers for *Pistacia* species and have been previously detected in several ancient Egyptian embalming materials^9,28,36^. However, in combination with dammarenolic acid, oleanonic acid is also a constituent of dammar resin from the Dipterocarpaceae family, among other angiosperm clades^45–47^. Apart from its natural occurrence in dammar resin, dammarenolic acid could also be an oxidation product of the compound dammaradienone, which is present in both dammar and Pistacia resin (Fig. 2e)^46,48^. Previous studies on mummification balms have also noted the overlap between compounds found in dammar and *Pistacia*^28,49^. Hence, based on current evidence, Pistacia and dammar resins cannot be unambiguously differentiated, and both resins are, therefore, considered possible sources for these compounds.

In addition to the terpenoids, phenolic and aromatic compounds were also detected in the balms, including vanillic acid, coumarin, and benzoic acid. Although vanillic acid is found in natural vanilla extracts, in this context it most likely reflects degradation of woody tissue^50–52^, and possibly derives from the conifers in the balm. It is more difficult to assign an origin to the aromatic compound coumarin, as it occurs naturally in a wide range of different and disparately related plants, among which are the cinnamons and many Fabaceae. Coumarin has a vanilla-like scent. Another aromatic compound—benzoic acid—was found in all samples. It also occurs in many plant gums and spices, such as gum benzoin, cinnamon and cloves or balsam type plants^32,53^. Given the ubiquity of these compounds in the plant kingdom, we could not assign them to a specific source.

### GC–MS and HT-GC–MS analysis

**Figure 3:**
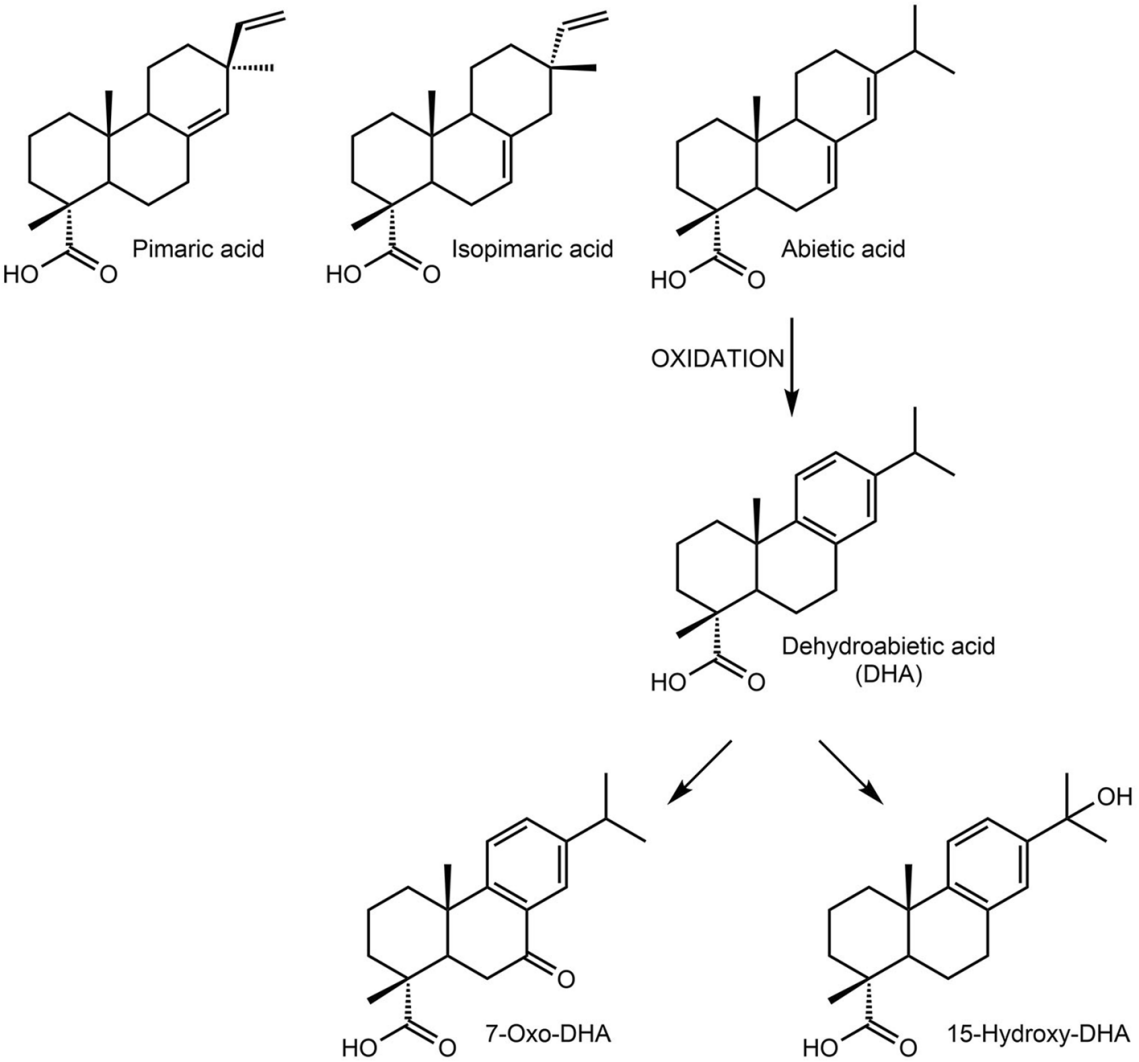
Resin acids present in samples from both canopic jars, and sequential oxidation reactions of abietic acid.

**Figure 4:**
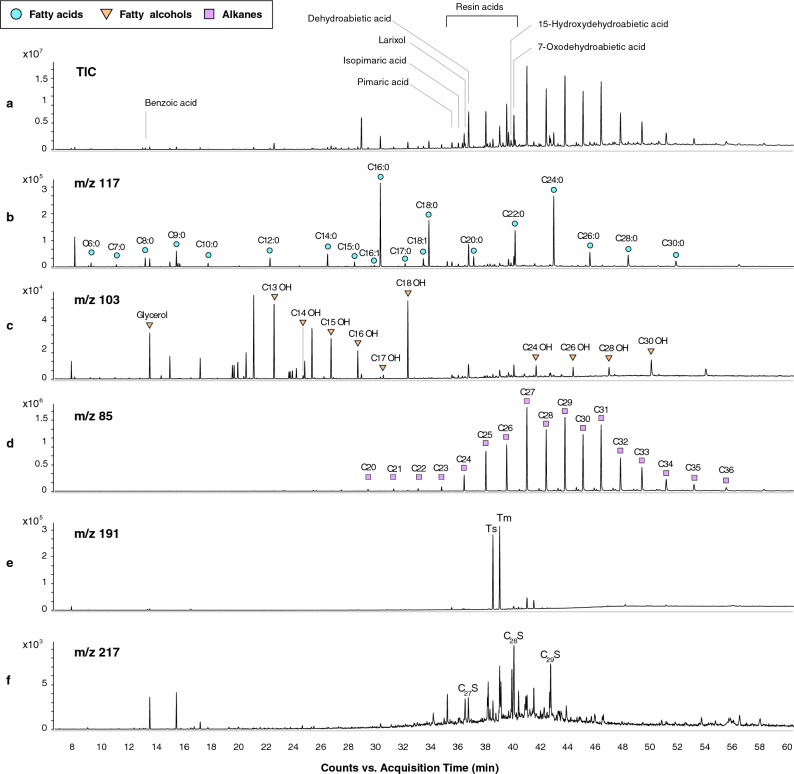
Total ion current (TIC, **a**) and extracted ion chromatograms (EIC, **b–f**) of sample AES 062 displaying ion masses of characteristic fragments from the main compound classes. (**b**) *m/z* 117 displaying fatty acids, n:0 = saturated FA and n:1 = unsaturated FA; (**c**) *m/z* 103 showing the distribution of fatty alcohols; (**d**) *m/z* 85 displaying *n*-alkanes with corresponding carbon numbers and (**e,f**) characteristic fragments of hopanes and steranes. For more detailed information of hopanes and steranes see Supplementary Figs. S4, S5. For identified compounds of samples AES 067 see Supplementary Fig. S2.

Additional GC–MS measurements of the lipid fraction were carried out to analyze fatty acids and alcohols, *n*-alkanes, and resin acids (Fig. 4a). Similar to the LC–MS/MS results, 7ODHA and DHA were detected in the lipid fraction together with abietic acid, pimaric acid, isopimaric acid and 15-hydroxydehydroabietic acid. The latter is another oxidation product that forms from abietic acid and DHA (Fig. 3). These resin acids were identified only in samples AES 062 and AES 067 taken from the bottom of the jars, but not in the remaining samples, which had an overall lower concentration of organic molecules (less than one tenth compared to AES 062 and AES 067 based on total peak area). All diterpenoids identified occur throughout all genera of Pinaceae, though the abundance of the primary resin acids pimaric acid and abietic acid is more characteristic of resins from *Pinus* species^34^. In addition to these biomarkers for Pinaceae resins, we also detected the compound larixol (Supplementary Fig. S6) in sample AES 062. Like the above-mentioned resin acids, larixol is also present in larch wood resin (*Larix* spp.)^54–57^, and is indeed specific to it. Its presence therefore suggests a *Larix* species as a possible source for the Pinaceae resin. However, this finding needs to be treated with caution, as it is based on a single biomarker. Additionally, we also cannot rule out a mixture of different Pinaceae genera, including both pine and larch resins.

Apart from the odiferous resins, the analysis of the lipidic fraction revealed that the balm contained additional ingredients (Fig. 4b). The profile was dominated by high abundances of saturated even-carbon-numbered straight-chain fatty acids, predominantly palmitic acid (C_16:0_) and lignoceric acid (C_24:0_) and, to a lesser extent, behenic acid (C_22:0_) and stearic acid (C_18:0_). These free fatty acids are end-products of the degradation of lipidic substances and can indicate a contribution of either plant oils, or animal/human fats^52,58,59^. The large number of very long-chain fatty acids (C_22:0_–C_30:0_) is characteristic of higher terrestrial plants and epicuticular waxes, as well as beeswax^60^. The odd-carbon-numbered straight-chain components detected in the samples [e.g., pentadecanoic acid (C_15:0_), and heptadecanoic acid (C_17:0_)] are sometimes seen as characteristic for ruminant lipids. However, their low abundance and the absence of corresponding branched isomers instead suggest that these compounds are more likely the result of bacterial degradation^61,62^. The samples also exhibit short-chain homologues from C_6:0_–C_10:0_, which are known to be degradation products caused by oxidation and formed during ageing or drying of organic tissue, for example of plant oil^28,59,63^. Monounsaturated fatty acids are also present in the form of octadecenoic acid (C_18:1_) and hexadecenoic acid (C_16:1_), which are found in vegetable oils and animal fats^52^. Thus, the fatty acid distribution suggests the balms most likely included a mixture of degraded animal fats and plant oils. Some caution in interpretation is required, however, since we cannot distinguish between fat that derives from an added animal ingredient or the human remains themselves.

Another class of compounds present in the lipid fraction was *n*-alkanes, which represent the most abundant compounds in sample AES 062. The extracts yielded medium and long chain *n*-alkanes, (C_20_–C_36_), displaying a slight odd-over-even predominance, with C_27_ as the most abundant *n*-alkane (Fig. 4d). Given the presence of this homologous series of *n*-alkanes, which is characteristic for fossil hydrocarbons, we hypothesized that the *n*-alkanes might reflect bitumen, a substance often associated with Egyptian mummification^28,64,65^. For this reason, we screened for characteristic hopanes and steranes of bitumen (ions *m/z* 191 and *m/z* 217; Fig. 4e, f and Supplementary Figs. S4 and S5). These ions are diagnostic markers for natural petroleum^8,66,67^, and were detected in the samples from both canopic jars, thus confirming the presence of bitumen.

**Figure 5:**
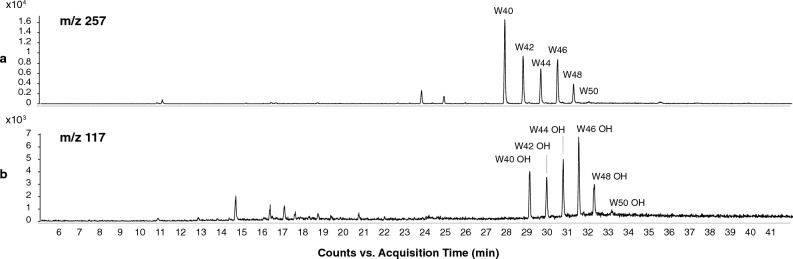
Extracted ion chromatograms (EIC) of HT-GC–MS analyses for *m/z* values 257 and 117 of sample AES 062 displaying monoesters of palmitic acid (**a**) and hydroxy palmitic acid esters (**b**).

*n*-Alkanes with a chain length from C_25_ to C_35_, but a strong odd-over-even dominance, are also known to be characteristic of epicuticular waxes of higher terrestrial plants^59,68,69^, and of beeswax, which has been reported in previous mummy balm studies^29,36,60^. Beeswax usually consists also of wax esters with a carbon chain length of greater than 40. The samples AES 062 and 067 were additionally analyzed by HT-GC–MS to search for these wax esters. We detected small amounts of monoesters of palmitic acid ranging from C_40_ to C_50_, as well as the corresponding hydroxy wax esters (Fig. 5) in both samples. The presence of the wax esters, the *n*-alkane distribution with the most abundant peak for C_27_, the long-chain fatty acids, and the *n*-alcohols (Fig. 4c) provide robust evidence for the use of beeswax as prominent ingredient in the balms.

## Discussion

Our analysis shows that rich information is recoverable from the remnants of balms in Egyptian canopic jars, even when such jars have been emptied and transferred between museum collections for more than a century. The samples taken from Senetnay’s jars provide evidence for incorporation of a variety of natural products and odiferous ingredients in the balms used to preserve her organs. Oils and fats, together with beeswax and bitumen, seem to have formed the basis of the balms identified in both jars, and our analysis demonstrates that these substances were mixed with coniferous resins, specifically from Pinaceae. Additionally, our analyses revealed the presence of other unidentified plant products containing benzoic acid and coumarin. Previous analyses of other Egyptian balms have also observed benzoic acid, together with phenolic acids, which have been associated with the presence of aromatic plant exudates of balsamic resins or gums^9,10^.

Analysis further revealed that the balms from Senetnay’s two jars were not identical in composition. The balm of canopic jar 1, which originally contained Senetnay’s lungs, included an additional aromatic resin (probably dammar or *Pistacia* resin) that was not found in jar 2 (which contained her mummified liver). Additionally, the compound larixol, suggestive of larch resin, was only detected in jar 1. Apart from these ingredients, the composition of the balms in the two jars appear to have been very similar, although the ratios of the ingredients in each is different. The differences in the balms chemical composition might suggest that balms were organ-specific, highlighting the importance of in-depth investigations of balms from canopic jars. However, given the fact that the samples from Senetnay’s canopic jars are almost 3500 years old, and multiple degradation processes likely occurred over the period of deposition and storage, we cannot exclude the possibility that the resinous ingredients were originally the same but have degraded differently through time. Additionally, it is possible that the mummification balm was heterogeneous and that ingredients were not thoroughly mixed or evenly distributed. Nevertheless, we find some support for the notion of organ-specific balm recipes from a recent study of inscribed vessels for the preparation of embalming materials from a mummification workshop at Saqqara, dating to the mid-first millennium BCE^31^. In the Saqqara example, the different mixtures were not found in canopic jars, but rather in vessels in which mummification balms were being prepared for later application to the liver and stomach. In contrast, our study analyzed balms deriving from already embalmed organs and our results provide tentative support for the hypothesis that different balms were applied to different organs.

**Figure 6:**
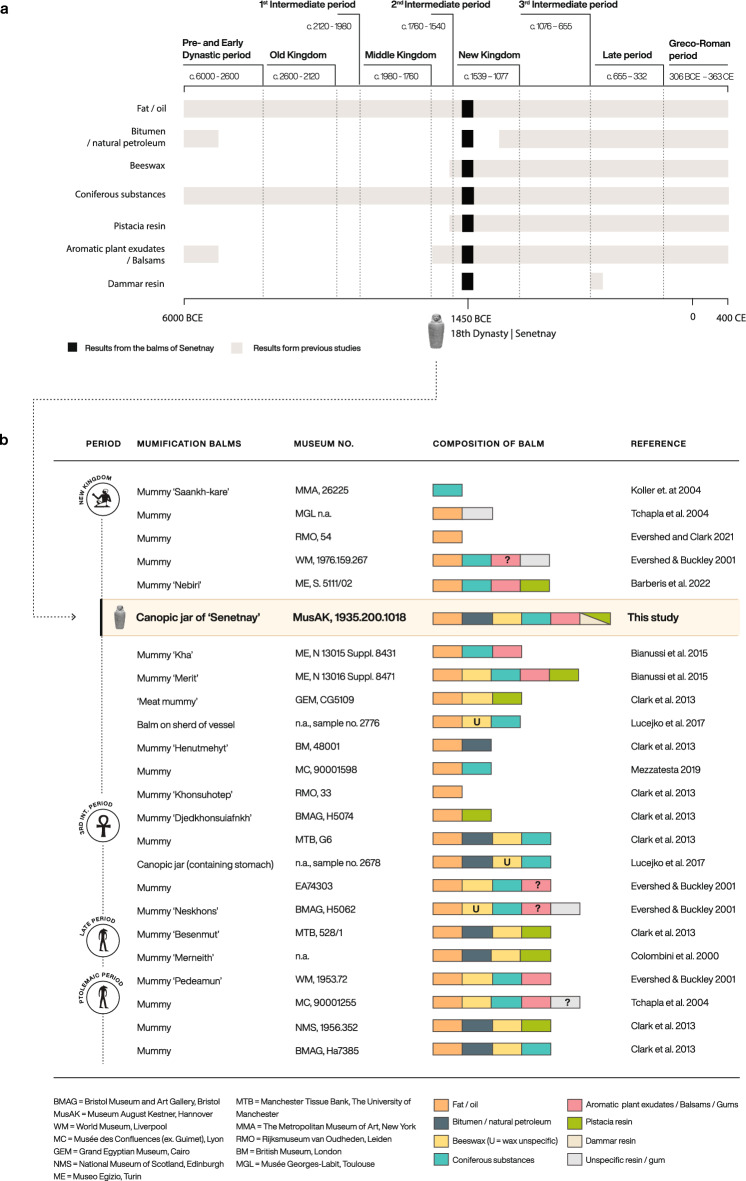
(**a**) Occurrence of reported substances in Egyptian balms through time (sources:^8,9,24,26–29,31,32,35–38,40,41,49,62,70–74^). (**b**) Composition of mummification balms from the New Kingdom, contemporary to Senetnay, and selected balms from the Third Intermediate Period to the Ptolemaic period consisting of 4 or more substances.

Our review of the literature on previous balm analyses shows that some of the ingredients we find in the mummification balms used on Senetnay’s organs (e.g., bitumen) were not commonly used for embalming in New Kingdom Egypt. Previous analyses suggests that ancient Egyptian mummification balms contained a limited range of ingredients before the Third Intermediate Period (c. 1000 BCE), becoming more complex through time^24^. While analysis of very early balms has revealed the use of multiple ingredients^8^, Egyptian balms through the Old and Middle Kingdoms often consisted solely of fats or oils (Fig. 6A). Only in the Second Intermediate Period and New Kingdom (c. 1760–1077 BCE) did balms become more complex, with the introduction of diverse resins, likely reflecting both evolving approaches to mummification, and the increasing ability to acquire ingredients from further afield^24^. In general, in the mid-second millennium BCE, when Senetnay died, only a small number of mummies received this kind of elaborated treatment.

Other examples of sophisticated balms in this period come from the high-status Eighteenth Dynasty (ca. 1479–1424 BCE) burial of a dignitary named Nebiri^49^, as well as from the mummies of the royal architect Kha and his wife Merit^70^. When analyzed, these balms were found to contain fats and oils, coniferous resins, and aromatic plant products or gums. The balms from Nebiri and Merit additionally contained *Pistacia* resin and Merit’s embalming also had beeswax in it. Senetnay’s embalming, also from the Eighteenth Dynasty, and contemporary to or slightly younger than Nebiri’s burial, but earlier than those of Kha and Merit, featured another unique and distinctive balm. This included beeswax and fat/oil, as well as an aromatic or balsamic substance, together with coniferous resin (possibly larch resin), and *Pistacia* resin or even a very exotic component in the form of dammar resin. Additionally, Senetnay’s balms also contained bitumen, which is evidence of very early use of this natural substance in the context of mummification. Chemical analyses have not yielded any other example of such a complex balm with 6 ingredients (in jar 1) in the mid-second millennium BCE in Egypt (Fig. 6B). Beeswax and bitumen also only became major ingredients of mummification balms towards the end of the New Kingdom. Overall, the balms used in Senetnay’s jars contain ingredients that were commonly employed in Egypt only in later periods, particularly at the “height of mummification” in the first millennium BCE, when balms became more complex and elaborated. Senetnay’s balm might therefore be seen as a forerunner for a later trend. It is important to note, however, that the increased number of ingredients identified in Senetnay’s balm might simply reflect better preservation and/or our multi-analytical approach, which involved the combined use of GC–MS, HT-GC–MS, and LC–MS/MS, allowing for a more holistic approach to the study of the balm samples. While Fig. 6 synthesizes data from a range of studies, the sample preparation and analytical approaches of previous analytical studies have varied and are not directly comparable. Bitumen in particular, is likely underrepresented due to the necessity of specialized procedures in sample preparation.

Notwithstanding these caveats, Senetnay’s remains seem to have received special treatment. The ingredients in her mummification balms give the impression of a woman of exceptional social standing, suggesting, along with other lines of evidence, that she was a highly valued member of the Pharaoh’s entourage. The elaborate treatment of Senetnay’s remains is echoed in the broader pattern of her burial. Her very presence in the Valley of the Kings, a necropolis normally reserved for pharaohs and powerful nobles^75^, points to extraordinary privilege, and the high regard in which Senetnay was likely held by the Pharaoh. Her title, “Ornament of the King”^17^, further reinforces the evidence for her special standing.

**Figure 7:**
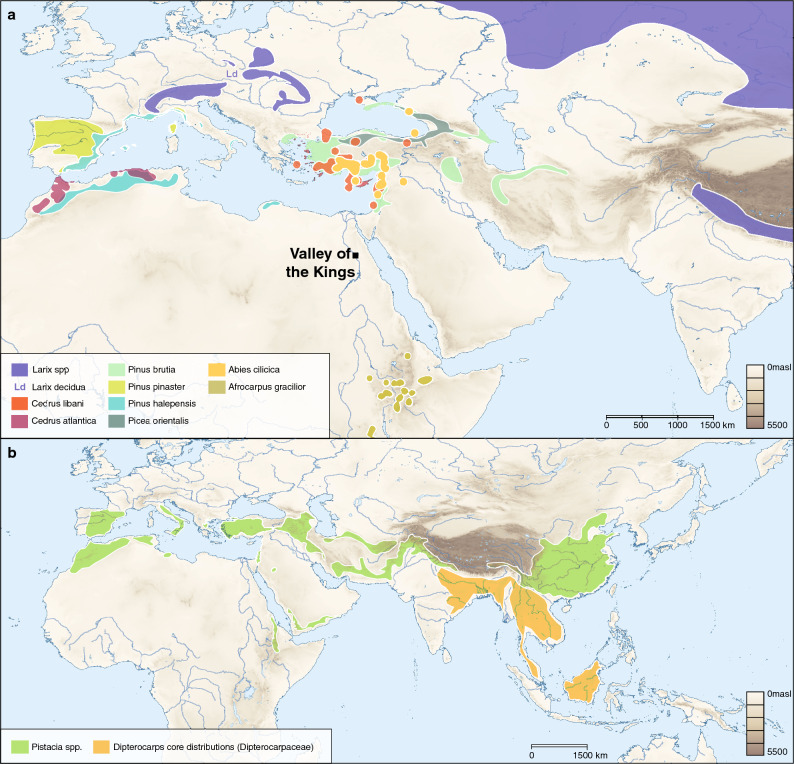
(**a**) Map showing the distribution of potential conifer resin sources in relation to the Valley of the Kings. (**b**) Map displaying the natural habitat of *Pistacia* spp. and the core distribution of Dipetrocarpus and Hopea (Dipterocarpaceae family), excluding small population in the Western Ghats of South India. Conifer and Dipterocarpaceae distributions are based on various sources (see Supplementary Table S6). The maps were created using QGIS 3.12 (https://qgis.org/en/site) and use Natural Earth vector map data from (https://www.naturalearthdata.com/downloads/).

In keeping with these indications of a woman of prominent status are the origins of the ingredients in the balms employed in Senetnay’s canopic jars. Most of the ingredients in her balms were of non-local origin, and, thus, depended on transport to be available in Egypt. Trees of the pine family, for example, are not endemic to Egypt (Fig. 7a). As noted, one possible Pinaceae resin source is larch wood resins from *Larix* species, based on our finding of the compound larixol. Larch resin has also been identified in historical medical remedies in Rome on the basis of the presence of the compound larixol^76^. There are ten recognized species in the *Larix* genus, of which only one is native to Europe (*L. decidua*)^77^, while none are native to southwest Asia or Africa^78^. While there are species native to Siberia (*L. sibirica; L. gmelinii)*, and such South Asian mountain chains as the Himalayas (*L. potaninii; L. mastersiana, and L. griffithii*), these are much further from Egypt and thus less plausible sources for the resin in this study. *L. decidua* exists in mountain-top refugial populations across the Pyrenees, Alps, and other western Mediterranean and Central European mountains, and could have been obtained via sea trade, though putative Egyptian trade contacts with Central Europe are poorly understood at present^79^.Other Pinaceae sources are also possible though, and those near the ancient Egyptian realm could have included the Cilician fir (*Abies cilicia*), Lebanese and Atlas cedar (*Cedrus libani* and *atlantica*), Asian spruce (*Picea orientalis*), Aleppo pine (*Pinus halepenis*) and the parasol pine (*P. pinea*). The Turkish pine (*P. brutia*) and maritime pine (*P. pinaster*) grow further north in the Mediterranean, notably on many islands and in northern coastal areas. While there is some evidence for population shifts among some of these conifers, notably mid-Holocene range reduction^80^, there is no reason to believe that there are any species that existed in Egypt over the past three millennia but are no longer present. Coniferous ingredients within the balms are, therefore, most likely imported products.

Apart from coniferous resins, our analysis also points to the presence of another aromatic plant exudate, which might be either *Pistacia* or dammar resin. *Pistacia* trees, notably *P. terebinthus* and *P. lentiscus*, are native to the Mediterranean coastal region, ranging from southern Spain to the Levant (Fig. 7B). Both of these species have a long history of use for their resins, producing turpentine and mastic resins, respectively. Beyond their use in ancient Egypt^81^, later Classical sources show how widely these resins were used across the Mediterranean^82^. Tree species that produce dammars (primarily in Dipterocarpaceae), meanwhile, grow exclusively in southeast Asian tropical forests^83^. Evidence for this type of exotic gum resin is thus unexpected and has not been reported in ancient Egyptian mummification balms from the second millennium BCE. If confirmed, the presence of dammar resin, which has recently been identified in balms from Saqqara, dating to the first millennium BCE^31^, would suggest that the ancient Egyptians had access to Southeast Asian resins that arrived in the Mediterranean by long-distant trade almost a millennium earlier. Some support for such long-distance links is perhaps indicated by the finding of peppercorns in the nostrils of the mummy of the pharaoh Ramses II, dated ca. 1200 BCE^84,85^. This spice is endemic only to the wet forests of southern India^86^. Even earlier African-Indian exchange is hinted at by the presence of crops of African origin in the Indian subcontinent by 2000 BCE, where they were being grown on Harappan farms^84^. Nonetheless, these early long-distance trade connections remain very poorly understood, with no associated material culture evidence, and *Pistacia* is the more parsimonious identification at present. If confirmed, this would represent one of the earliest direct identifications of *Pistacia* resin in a mummification balm. Apart from its appearance in the balm of Nebiri, *Pistacia* resin was also used in the preparation of “victual” or food mummies from the late Seventeenth-early Eighteenth Dynasty, when it was applied to some of their wooden coffinets and bandages^74^. Overall, the findings point to early evidence for trade in exotic plants and/or plant substances between Egypt and its near neighbors, with the possibility of early trade links that extended further afield.

Our analysis reveals rich information about social status, technological acumen, and trade that can be obtained from apparently empty archaeological jars excavated more than a century ago. It joins a growing number of studies that highlight the value of applying new methods to investigate trace remains and amorphous residues as well as long-held museum specimens^31,41,53,73,87–90^. Together with these other studies, our findings demonstrate that analytical chemistry is able to shed significant light on the identification of ingredients included in ancient balms, adding substantially to information recoverable from ancient textual sources. At the time that Senetnay’s viscera were discovered by Howard Carter, the methods that we have employed in this study would not have been imagined possible. Yet, over 120 years later, the royal tomb known as KV 42 and its contents continue to provide new information about ancient Egyptian cultural practices, society and trade. Our study thus highlights not only the invaluable role of science in archaeological research but also the importance of conserving cultural heritage under optimal conditions over the long term.

## Methods

### Sampling of ancient mummification balms

The samples of the mummification balm were collected from two ancient Egyptian limestone canopic jars at the Museum August Kestner in Hannover. The jars date to the Eighteenth Dynasty (1450 BCE) and hold the viscera of the noble lady Senetnay. While the jars were empty, a thin layer of organic residue was preserved at the bottom of each. Samples of the embalming material from canopic jar 1 (containing the lungs) and jar 2 (containing the liver) were collected from various parts of the jar (walls and bottom of the jars; see Supplementary Fig. S1). Before collecting these samples, a thin surface layer was removed at the specific sampling spots using disposable scalpels to avoid contamination. Subsequently, samples were taken from below the surface layer with a scalpel. From each spot, ca. 200 mg of residual crust was taken. This was not possible for the remains attached to the walls of the jars, as the layers were very thin and the residues were mostly preserved within the porous matrix of the limestone. In these cases, the residues were removed with a scalpel without first removing the surface layer to recover enough material (ca. 100–200 mg) for analysis. All samples were immediately placed in glass vials that were previously combusted at 500 °C for 8 h to remove potential contaminants, until further processing under clean lab conditions in the laboratory of the Max Planck Institute for the Science of Human History, Jena, Germany.

### Materials

Methanol (MeOH), dichloromethane (DCM), and methyl *tert*-butyl ether (MTBE) used for the analyses, as well as the analytical standards isopimaric acid and vanillic acid, were obtained from Sigma-Aldrich (Munich, Germany). In addition, 7-oxodehydroabietic acid was obtained from Campro Scientific (Berlin, Germany), dehydroabietic acid from Carbosynth (Berkshire, UK), pimaric acid from Abcam (Berlin, Germany), palustric acid from Toronto Research Chemicals (Toronto, Canada), neoabietic acid and oleanonic acid from Santa Cruz Biotechnology (Heidelberg, Germany), dammarenolic acid from Enzo Life Sciences (Lörrach, Germany), moronic acid from TCI chemicals (Eschborn, Germany), coumarin from LGC Standards (Wesel, Germany) and benzoic acid from Agilent Technologies (Frankfurt, Germany). MS-grade formic acid (FA) was purchased from VWR (Leuven, Belgium), while acetonitrile (ACN) and water used for HPLC–MS/MS analyses were purchased from Biosolve (Valkenswaard, Netherlands).

### Extraction and analysis

Samples were extracted following established protocols^91,92^, with modifications made for the extraction of ancient samples. Briefly, 50–100 mg of the sample were homogenized into a fine powder and solvent extracted using an MTBE: MeOH (3:1, v/v) extraction mixture. After vortexing the mixture and shaking for 45 min, the samples were ultrasonicated for 15 min. Subsequently, a H_2_O: MeOH (3:1, v/v) solution was added to each sample and mixed well again. The samples were then centrifuged at 20,000×*g* for 5 min. At this stage, a dense pellet of precipitated proteins formed on the bottom, as well as two liquid phases: (1) an upper phase containing the hydrophobic lipids, which form due to the low density of MTBE and (2) a lower phase with semi-polar and polar metabolites. Each of the two liquid phases were transferred separately to new glass vials, while the remaining pellet was washed with methanol and stored in a −80 °C freezer for future palaeoproteomic analysis, awaiting the development of more plant reference material in protein reference databases. Aliquots of samples with the lipid-containing phase were derivatized with 100 μL *N,O-*bis(trimethylsilyl)trifluoroacetamide (BSTFA, containing 1% TMCS, Sigma-Aldrich) for 60 min at 70 °C and then analyzed by GC–MS. The lower phase containing the polar metabolites was dried in a vacuum concentrator and re-suspended in HPLC-grade MeOH before LC–ESI–MS/MS analysis.

GC–MS analyses were performed using an Agilent 8890 GC-System coupled to an Agilent 5977B GC/MSD. Chromatographic separation was achieved on a HP-5ms 60 m × 250 μm capillary column (Agilent) with a film thickness of 0.25 μm. The mass spectrometer was operated in electron impact (EI) mode at 70 eV and helium was used as a carrier gas with a constant flow rate of 1.0 mL/min. The GC oven temperature was set at 60 °C and held for 2 min, then ramped to 120 °C at a rate of 30 °C/min and held for 2 min. The temperature was increased again at 5 °C/min to 320 °C with a final hold time of 15 min. The total run time was 61 min with a solvent delay of 6.5 min. Injection volume was 1 μL and a split ratio of 10:1 was used to improve peak shapes. The scanning range was set from *m/z* 30 to 700 amu. Injection blanks were carried out between each sample to avoid carryover. Transfer line and source temperature were set at 250 °C and 230 °C, respectively.

High temperature GC–MS analyses were performed on an Agilent 8860 GC coupled to a 5977B mass spectrometer. Samples (1 µL) were injected onto a DB-1HT column (15 m × 250 µm i.d., 0.1 µm film thickness) column using a cool-on-column injector. Helium was used as carrier gas with a constant flow rate of 1.2 mL/min. The GC oven was programmed as follows: After 2 min at 50 °C the temperature was increased to 350 °C at a rate of 10 °C/min. This final temperature was held for 10 min. The temperature of the transfer line, ion source and quadrupole were set to 350 °C, 230 °C and 150 °C, respectively, while the inlet temperature was set to track the oven temperature. Electron ionisation at 70 eV was used and data was recorded in full scan from *m/z* 50 to 800 amu after a solvent delay of 5 min.

LC–ESI–MS/MS analysis was carried out using a Shimadzu LCMS-8050 triple-quadrupole system. The HPLC was equipped with LC-30AD binary pumps, a DGU-20A5R solvent degasser, CTO-20AC column oven and a SIL-30AC auto sampler. Chromatographic separation was performed on a Shimadzu Shimpack Velox SP-C18 column (100 mm × 2.1 mm, 2.7 µm particle size) and a Restek Raptor Biphenyl analytical column (100 mm × 2.1 mm, 2.7 µm) particle size. The mobile phase consisted of HPLC grade H_2_O and 0.1% FA (mobile phase A) and ACN (mobile phase B). The column temperature was fixed at 25 °C and the gradient program was 0.5% B from 0–1 min, to 80% B at 10 min, to 100% B at 15 min with a hold until 17.5 min, and back to 0.5% B and held until 20 min. The solvent flow rate was maintained at 0.2 mL/min for analyses using the C18 column and 0.3 mL/min for analyses with the biphenyl column, and injection volumes were set at 1 or 2 µL (depending on sample concentration). Ionization was performed with an electro spray ionization (ESI) ion source with detection in both positive and negative modes. All samples were analyzed in duplicates.

Data processing and analysis of GC–MS data was performed using the Agilent MassHunter Qualitative Data Analysis software 10.0. Peak identification was carried out based on comparison with retention times and mass spectra of analytical standards where available, by comparison to the reference mass spectral library NIST (2.2), and with spectra reported in the literature. LC–MS/MS data were collected and processed using LabSolutions software (Shimadzu, Kyoto, Japan). The multiple reaction monitoring (MRM) mode was used for analysis, with authentic analytical standards for the optimization of MRM parameters employed to screen for specific compounds in archaeological samples (see Supplementary Table S3 for list of all compounds, and Table S4 for MRM parameters).

## Supplementary Information

Supplementary Information.

Supplementary Table S4.

Supplementary Table S6.

Supplementary Table S7.

## Data Availability

All data generated or analysed during this study are included in this published article and its supplementary information files. For any additional information, please contact Barbara Huber (huber@gea.mpg.de).
