# Supplementary material for: Biomolecular characterization of 3500-year-old ancient Egyptian mummification balms from the Valley of the Kings
Source: Sci Rep. 2023 Aug 31;13:12477. doi: 10.1038/s41598-023-39393-y (PMC10471619; doi:10.1038/s41598-023-39393-y)
Supplement: Supplementary file 1 — Supplementary Information. [file 41598_2023_39393_MOESM1_ESM.docx]

**Biomolecular characterization of 3500-year-old ancient Egyptian mummification balms from the Valley of the Kings**

Huber, B., Hammann, S., Loeben, C. E., Jha, D. K., Vassão, D. G., Larsen, T., Spengler, R.N., Fuller, D. Q., Roberts, P., Devièse, T., Boivin, N.

Content

[Supplementary Figure S1 2](#_Toc138760651)

[Supplementary Table S1 2](#_Toc138760652)

[Supplementary Figure S2 3](#_Toc138760653)

[Supplementary Figure S3 4](#_Toc138760654)

[Supplementary Figure S4 5](#_Toc138760655)

[Supplementary Figure S5 5](#_Toc138760656)

[Supplementary Figure S6 7](#_Toc138760657)

[Supplementary Table S2 8](#_Toc138760658)

[Supplementary Table S3 9](#_Toc138760659)

[Supplementary Table S5 11](#_Toc138760660)

# Supplementary Figure S1

***
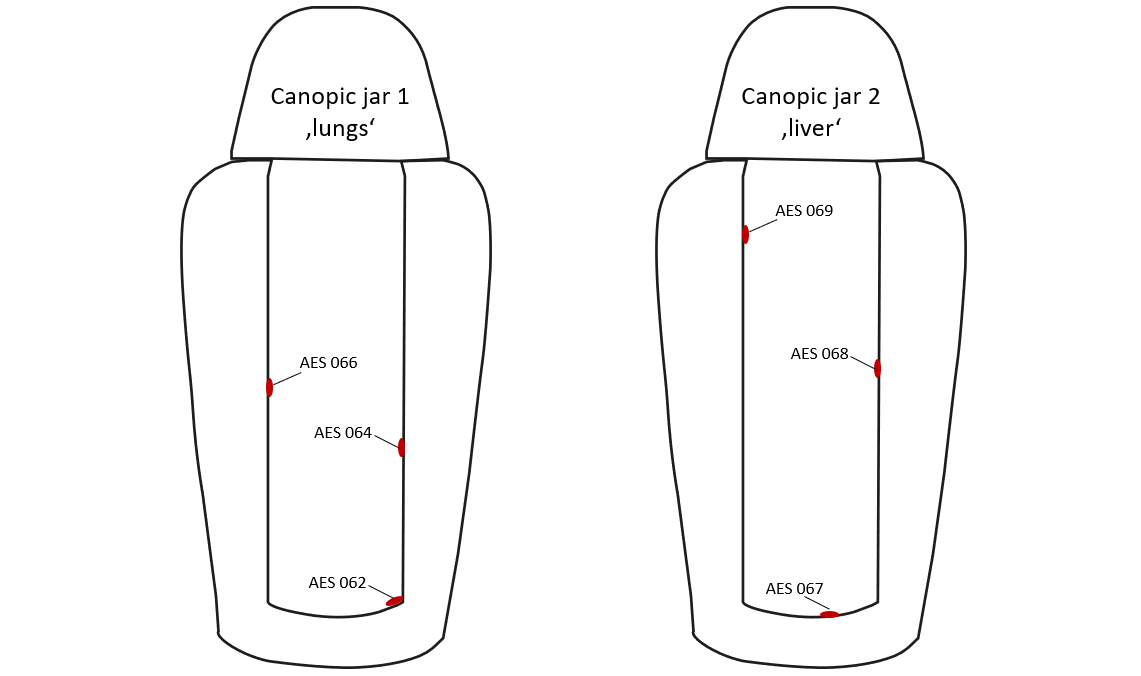
***

Schematic drawings of Senetnay’s canopic jars 1 and 2 from the August Kestner Museum, Hannover, Germany. The red dots indicate the location from which each sample was taken.

# Supplementary Table S1

| ***Lab sample no.*** | ***Sample location*** | ***Description of sample*** | ***Object no.*** | ***Dating*** | ***Context*** |
| --- | --- | --- | --- | --- | --- |
| DA-AES 062 | Interior, bottom of jar | Black, thin remnant of balm on the bottom of the jar | Acq. No. 1935.200.1018, Canopic jar of Senetnay (lungs) | New Kingdom, c. 1450 BCE | Tomb 42, Valley of the Kings, West Thebes |
| DA-AES 064 | Interior, wall of jar | Thin black spot of residue, partially absorbed within the porous material of the limestone | Acq. No. 1935.200.1018, Canopic jar of Senetnay (lungs) | New Kingdom, c. 1450 BCE | Tomb 42, Valley of the Kings, West Thebes |
| DA-AES 066 | Interior, wall of jar | Thin black spot of residue, partially absorbed within the porous material of the limestone | Acq. No. 1935.200.1018, Canopic jar of Senetnay (lungs) | New Kingdom, c. 1450 BCE | Tomb 42, Valley of the Kings, West Thebes |
| DA-AES-067 | Interior, bottom of jar | Black residual spot in the middle of the bottom of the jar | Acq. No. 1935.200.0253, Canopic jar of Senetnay (liver) | New Kingdom, c. 1450 BCE | Tomb 42, Valley of the Kings, West Thebes |
| DA-AES 068 | Interior, wall of jar | Thin black spot of residue, partially absorbed within the porous material of the limestone | Acq. No. 1935.200.0253, Canopic jar of Senetnay (liver) | New Kingdom, c. 1450 BCE | Tomb 42, Valley of the Kings, West Thebes |
| DA-AES 069 | Interior, wall of jar | Thin black spot of residue, partially absorbed within the porous material of the limestone | Acq. No. 1935.200.0253, Canopic jar of Senetnay (liver) | New Kingdom, c. 1450 BCE | Tomb 42, Valley of the Kings, West Thebes |

The table provides a description of the individual samples with all relevant information regarding the museum objects from which they derive.

# Supplementary Figure S2


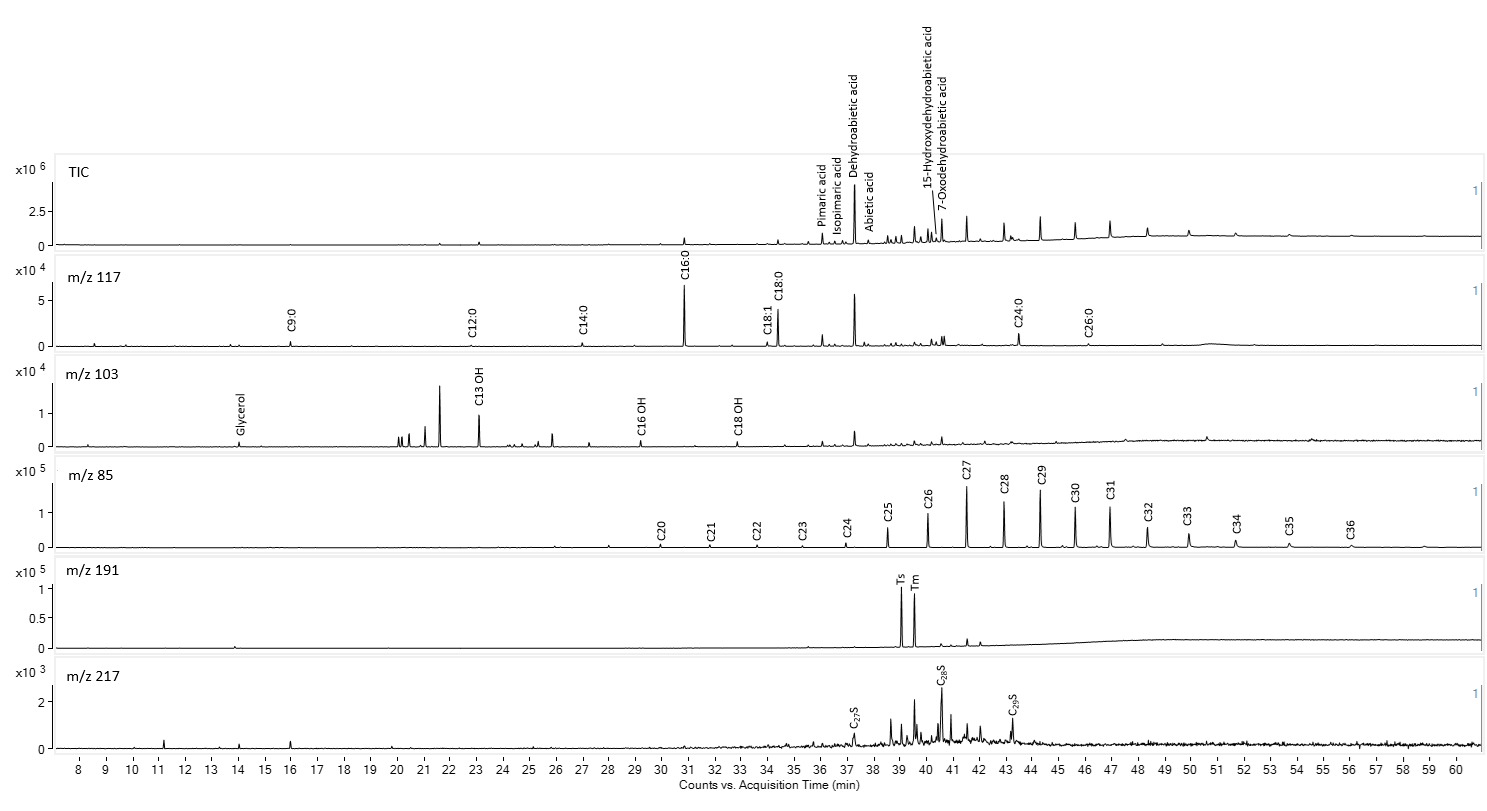


Total ion current (TIC) and extracted ion chromatograms (EIC) of sample AES 067 displaying fatty acids (*m/z* 117; n:0 = saturated FA and n:1 = unsaturated FA), fatty alcohols (*m/z* 103), n-alkanes (*m/z* 103), hopanes (*m/z* 191) and steranes (*m/z* 217). For detailed identification of hopanes and steranes in this sample see Supplementary Figure S5.

# Supplementary Figure S3


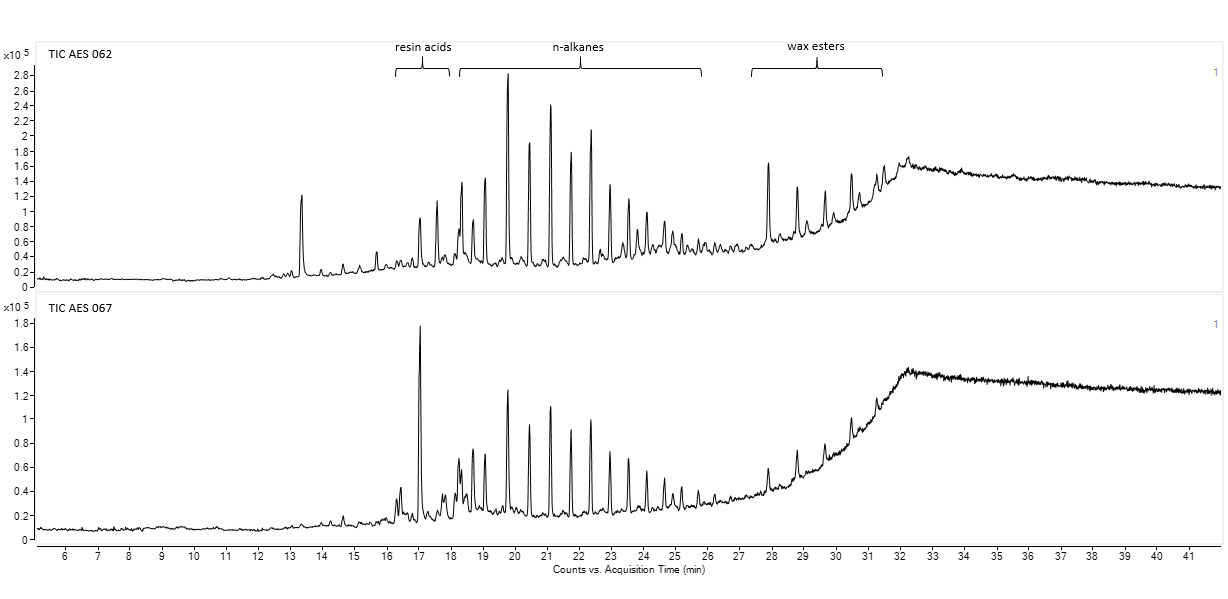


Additional TIC chromatograms of samples AES 062 and AES 067 obtained by HT-GC-MS showing the presence of resin acids, *n*-alkanes and wax esters. For detailed identification of the individual monoesters of palmitic acid and hydroxy wax esters see Figure 5.

# Supplementary Figure S4


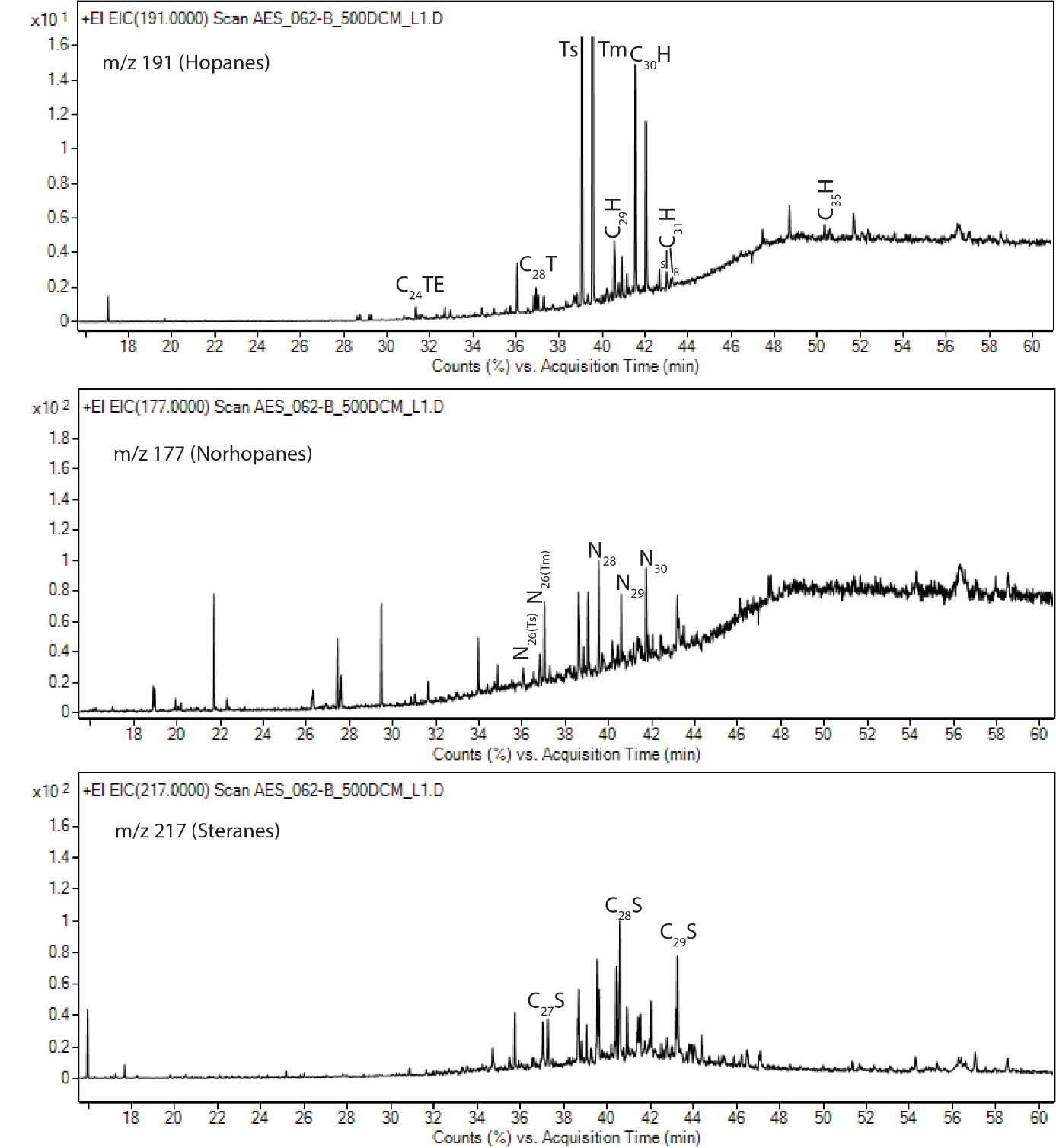


The hopane and sterane compounds identified in the sample AES 062. Their presence indicates use of bitumen in the balm. The presence of norhopanes (*m/z* 177) in the sample suggests degradation of these compounds. (The compounds are identified using parent and daughter ions (*m/z*) in EIC mode and some compounds were validated in SIM mode in GCMS). For the names of compounds, please see the Supplementary Table S2. Some compounds, which are crucial for understanding the provenance of the bitumen, were only present in low concentrations ^1–4^. Therefore, we omitted the ratio and provenance discussion to prevent potential inaccuracies in conclusions.

# Supplementary Figure S5


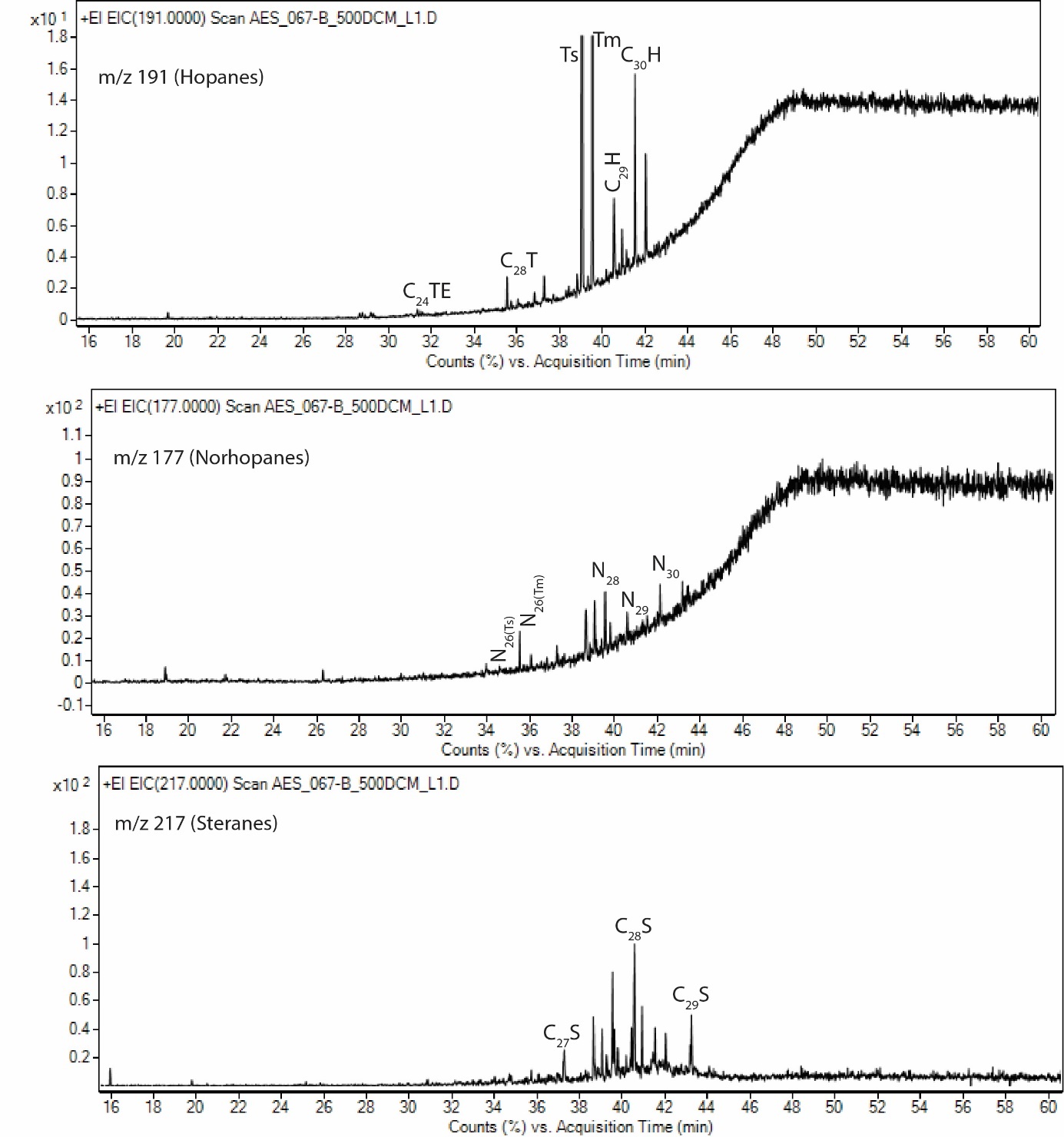


The hopane and sterane compounds identified in the sample AES 067. The compounds are similar to those identified in sample AES 062, but less in abundance. For the names of compounds, please see the Supplementary Table S2.

# Supplementary Figure S6

a


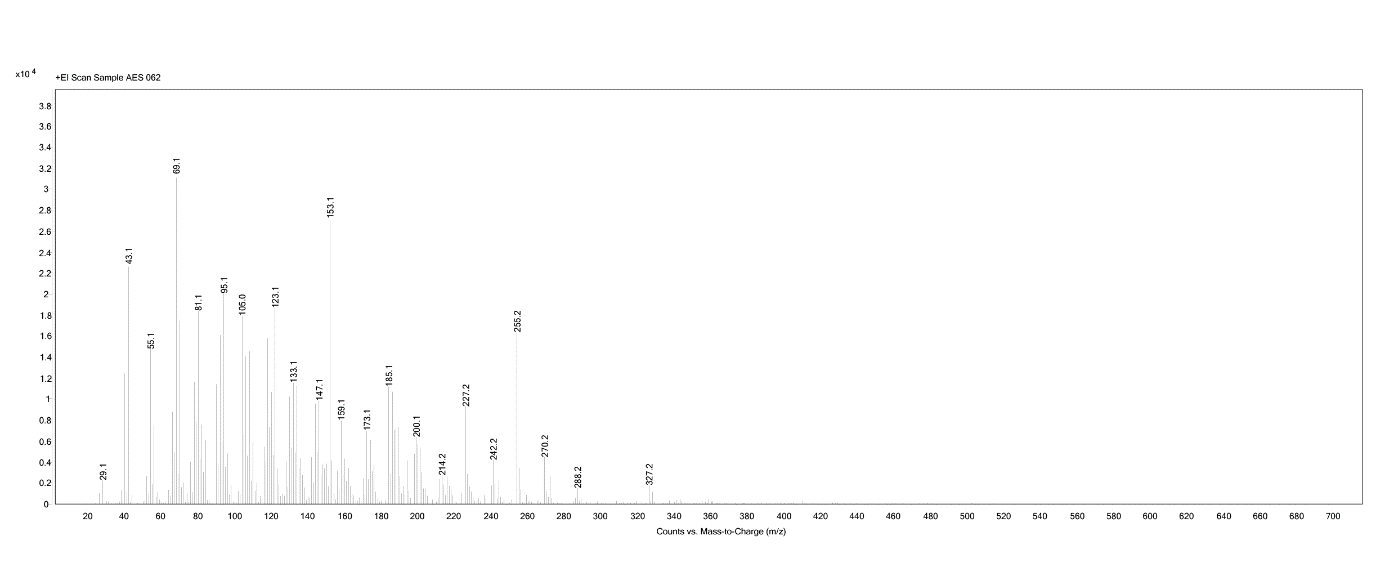


b


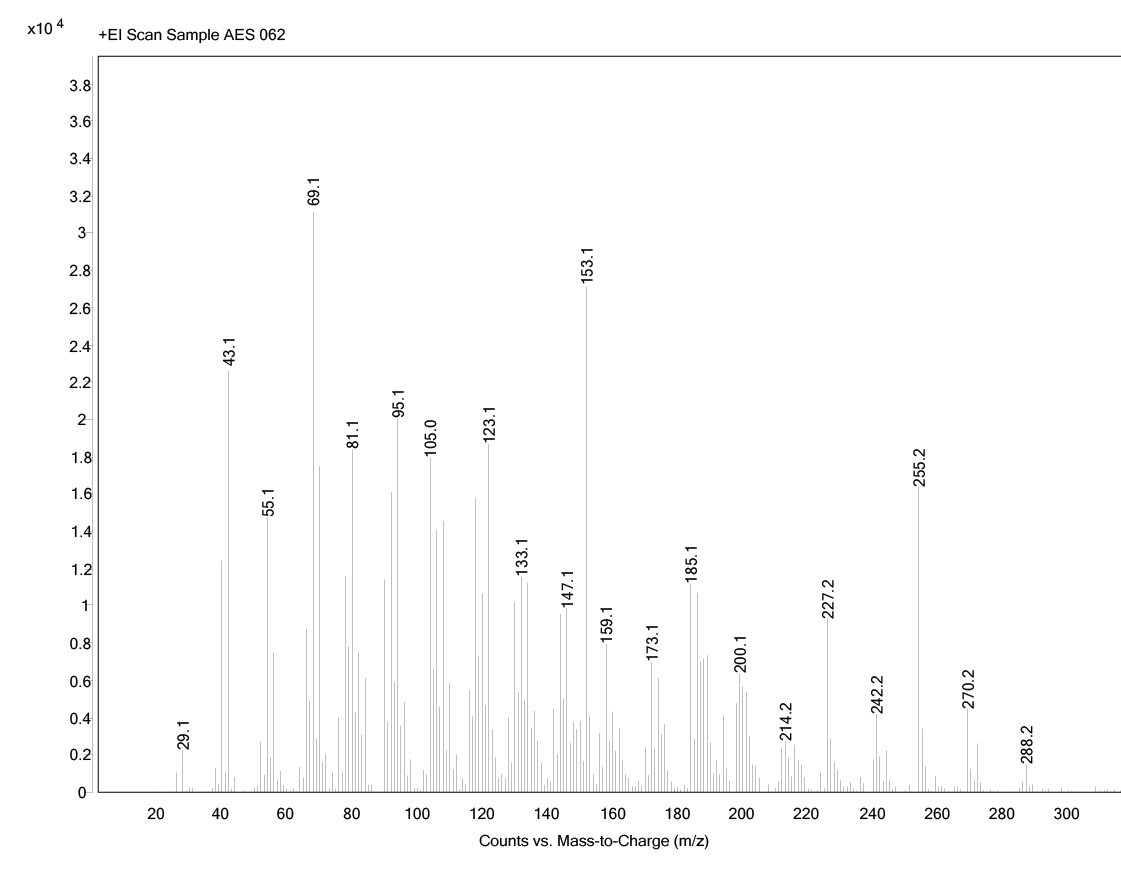


(a) Mass spectrum of the compound (rt: 37,017) identified as larixol and (b) partial mass spectrum of the same compound with more details.

# Supplementary Table S2

List of hopane and sterane compounds identified in samples AES 062 and AES 067. The identification was based on the combination of parent and daughter ions (*m/z*) extracted from the TIC. The ions were also verified using selected ion monitoring (SIM) method.

| **Compounds** | **Taget ions** | |
| --- | --- | --- |
|  | **Precursor (*m/z*)** | **Product (*m/z*)** |
| **Tricyclic terpanes (C28 T)** | 388 | 191 |
| **Tetracyclic terpane (C24 TE)** | 330 | 191 |
| **Regular 17α-hopane series** |  |  |
| 18α(H),21β(H)-22,29,30-trisnorhopane (Ts) | 370 | 191 |
| 17α(H),21β(H)-22,29,30-trisnorhopane ('Tm) | 370 | 191 |
| C29 Hopanes (C29 H) | 398 | 191 |
| C30 Hopanes (C30 H) | 412 | 191 |
| C31 Homohopanes (C31 H) | 426 | 191 |
| C35 Homohopanes (C35 H) | 482 | 191 |
| **Norhopane series** |  |  |
| N26 Norhopanes (Ts and Tm) | 388 | 177 |
| N28 Norhopanes (N28) | 384 | 177 |
| N29 Norhopanes (N29) | 398 | 177 |
| N30 Norhopanes (N30) | 412 | 177 |
| **Steranes** |  |  |
| C27 steranes (C27 S) | 372 | 217 |
| C28 steranes (C28 S) | 386 | 217 |
| C29 steranes (C29 S) | 400 | 217 |

# Supplementary Table S3

List of authentic analytical standards for the optimization of MRM parameters employed to screen for specific compounds in archaeological samples (for more detailed MRM parameters, such as collision energy (V) and dwell times for precursor and product ions see Supplementary Table S4 – separate excel file).

| **Compound** | **Column** | **Ret. Time (min)** |
| --- | --- | --- |
| Amygdalin | Velox SP-C18 | 5.834 |
| Anabasine | Velox SP-C18 | 1.573 |
| Artemisinin | Velox SP-C18 | 10.677 |
| Asiatic Acid | Velox SP-C18 | 9.967 |
| Benzoic Acid | Velox SP-C18 | 7.418 |
| Betulinic Acid | Velox SP-C18 | 14.410 |
| Caffeine | Velox SP-C18 | 6.097 |
| Cinnamic Acid | Velox SP-C18 | 8.217 |
| Cotinine | Velox SP-C18 | 5.158 |
| Coumarin | Velox SP-C18 | 8.069 |
| Curcumin | Velox SP-C18 | 10.065 |
| Demethoxycurcumin | Velox SP-C18 | 9.935 |
| Didemethoxycurcumin | Velox SP-C18 | 9.799 |
| Ferulic Acid | Velox SP-C18 | 6.830 |
| (E)-Guggulsterone | Velox SP-C18 | 11.676 |
| (Z)-Guggulsterone | Velox SP-C18 | 12.176 |
| Harmaline | Velox SP-C18 | 6.405 |
| Harmane | Velox SP-C18 | 6.157 |
| Harmine | Velox SP-C18 | 6.449 |
| Hydrocotarnine | Velox SP-C18 | 5.935 |
| Incensole | Velox SP-C18 | 13.904 |
| Meconic Acid | Velox SP-C18 | 5.240 |
| Nicotine | Velox SP-C18 | 1.560 |
| Nicotinic Acid | Velox SP-C18 | 1.564 |
| Oleanolic Acid | Velox SP-C18 | 14.647 |
| Opianic Acid | Velox SP-C18 | 6.530 |
| Quinine | Velox SP-C18 | 5.978 |
| Theobromine | Velox SP-C18 | 5.553 |
| ar-Turmerone | Velox SP-C18 | 12.066 |
| Vanillic Acid | Velox SP-C18 | 6.221 |
| Zingerone | Velox SP-C18 | 7.475 |
| Resin Acids (pimaric acid, isopimaric acid, palustric acid and neoabietic acid) | Biphenyl | 10.400 |
| 7-Oxodehydroabietic Acid | Biphenyl | 9.411 |
| Dehydroabietic Acid | Biphenyl | 10.091 |
| α-Boswellic Acid | Biphenyl | 11.101 |
| β-Boswellic Acid | Biphenyl | 11.296 |
| Acetyl α-Boswellic Acid | Biphenyl | 11.935 |
| Acety β-Boswellic Acid | Biphenyl | 12.159 |
| Keto β-Boswellic Acid | Biphenyl | 10.414 |
| Acetyl Keto β-Boswellic Acid | Biphenyl | 11.309 |
| Cholesterol | Biphenyl | 12.292 |
| Campesterol | Biphenyl | 12.493 |
| β-Sitosterol | Biphenyl | 12.727 |
| Brassicasterol | Biphenyl | 12.378 |
| 5α-Cholestanol | Biphenyl | 12.544 |
| Stigmasterol | Biphenyl | 12.681 |
| Sitostanol | Biphenyl | 12.998 |
| Cholestanone | Biphenyl | 13.376 |
| α-Amyrin | Biphenyl | 12.871 |
| β-Amyrin+Lupeol | Biphenyl | 12.620 |
| Benzoic Acid | Biphenyl | 6.141 |
| Ferulic Acid | Biphenyl | 5.876 |
| Dammarenolic Acid | Biphenyl | 10.723 |
| Masticadienolic Acid | Biphenyl | 10.912 |
| Moronic+Oleanonic Acids | Biphenyl | 11.010 |
| Dipterocarpol | Biphenyl | 11.475 |
| Urs-12-en-3-one | Biphenyl | 13.538 |

# Supplementary Table S5

Multiple reaction monitoring (MRM) chromatograms of the analytical standards neoabietic acid, palustric acid, pimaric acid and isopimaric acid summarized as ‘resin acids’.

| Neoabietic acid    Palustric acid    Pimaric acid    Isopimaric acid   |
| --- |
