# Supplementary material for: Biomolecular characterization of 3500-year-old ancient Egyptian mummification balms from the Valley of the Kings
Source: Sci Rep. 2023 Aug 31;13:12477. doi: 10.1038/s41598-023-39393-y (PMC10471619; doi:10.1038/s41598-023-39393-y)
Supplement: Supplementary file 3 — Supplementary Table S6. [file 41598_2023_39393_MOESM3_ESM.docx]

# Supplementary Table S6

Sources of occurrence data for candidate conifers (Fig. 7, A)

| **Taxon** | **Plants of the World Online [POWO; kew.org]** | **Farjon 2017; and other sources** | **GBIF.org (04 February 2023)** |
| --- | --- | --- | --- |
| *Larix decidua* Mill. | Austria, Czechoslovakia, France, Germany, Italy, Poland, Romania, Switzerland, Ukraine, Yugoslavia | Europe: Alps, Carpathians, Slovenian moutnains, S. Poland (Wista river); see also Karlman (2010). | <https://doi.org/10.15468/dl.pz45jj> [but includes many planted populations] |
| *Abies cilicica* (Antoine & Kotschy) Carrière | Lebanon-Syria, Turkey | Turkey: Anatalya and Konya (Isaurian Taurus) | https://doi.org/10.15468/dl.f59dkt |
| *Afrocarpus gracilior* (Pilg.) C. N. Page | Ethiopia, Kenya, Sudan, Tanzania, Uganda | Ethiopia, Kenya, South Sudan, Tanzania, Uganda | https://doi.org/10.15468/dl.uuyyxh |
| *Cedrus libani* A.Rich. | Cyprus, Lebanon-Syria, Turkey | Lebanon, Syria (Djebel el ANsiriya), Turkey (Taurus and Anti-Taurus mountains), Cyprus (Troodos Mountains, Mt. Triphylos) | https://doi.org/10.15468/dl.fvks6u |
| *Cedrus atlantica* (Endl.) Manetti ex Carrière | Algeria, Morocco | Algeria, Morocco (Atlas Mountains) | https://doi.org/10.15468/dl.gsk6mt |
| *Juniperus excelsa* M.-Bieb., | Albania, Bulgaria, Cyprus, Greece, Krym, Lebanon-Syria, North Caucasus, Palestine, Transcaucasus, Turkey, Yugoslavia | SE Europe, Central Asia, Middle East, Northwest India (Kashmir), Oman, Leabnan, Syria, Turkey, Cyprus |  |
| *Juniperus procera* Hochst. Ex Endl. | Djibouti, Eritrea, Ethiopia, Kenya, Malawi, Saudi Arabia, Somalia, Sudan, Tanzania, Uganda, Yemen, Zaïre, Zimbabwe | Ethiopia, Eritrea, Sudan (Red Sea hills), Somalia Yemen, Saudi Arabia (Asir mountains), elsewhere in tropical Africa (Tanzania, Uganda, NE Zimbabwe, Malawi, Congo) | https://doi.org/10.15468/dl.zs9qha |
| *Juniperus indica* Bertol | China South-Central, East Himalaya, Nepal, Pakistan, Tibet, West Himalaya | N Pakistan, Kashmir, Himalayas, from Himachal Pradesh eastwards into Yunnan and SW Sichuan | https://doi.org/10.15468/dl.ke9r9h |
| *Picea orientalis* (L.) Peterm. | North Caucasus, Transcaucasus, Turkey | Caucasus, North Turkey: coastal mountains | https://doi.org/10.15468/dl.gsk6mt |
| *Pinus halepensis* Mill. | Albania, Algeria, Baleares, Corse, East Aegean Is., France, Greece, Italy, Lebanon-Syria, Libya, Morocco, Palestine, Sardegna, Sicilia, Spain, Tunisia, Turkey, Yugoslavia | Mediterranean: From Morocco and Spain to Greece, Israel, Lebanon, SW Syria, Libya: Jabal al Akhdar; also, Critchfield and Little (1966). | https://doi.org/10.15468/dl.vh5hju |
| *Pinus brutia* Ten. | Bulgaria, Cyprus, East Aegean Is., Greece, Iran, Iraq, Kriti, Krym, Lebanon-Syria, North Caucasus, Transcaucasus, Turkey, Turkey-in-Europe | East Mediterranean, circum-Black sea, Turkey: Mugla Province Caucasus, NW Iran, N Iraq, Azerbaijain, Georgia, Afghanistan; also, Critchfield and Little (1966). | https://doi.org/10.15468/dl.n47ghf |
| *Pinus pinea* L. | Albania, Baleares, Corse, Cyprus, East Aegean Is., France, Greece, Italy, Kriti, Lebanon-Syria, Portugal, Sicilia, Spain, Turkey | Mediterranean Europe and Near East, original native range unclear, but Critchfield and Little (1966) infer a native range only on the Iberian Peninsula) | <https://doi.org/10.15468/dl.xpv569>  [but includes many planted, and anthropogenic populations] |
| *Pinus pinaster* Aiton | Algeria, Baleares, Corse, France, Italy, Morocco, Portugal, Sardegna, Sicilia, Spain, Tunisia | Western Mediterranean: France, Spain, Baleraic islands, Italy, Malta; ; also, Critchfield and Little (1966). | https://doi.org/10.15468/dl.taxaw9 |

Sources:

Farjon, Aljos (2017) *A Handbook of the World’s Conifers.* Leiden: Brill; GBIF= <https://www.gbif.org/> ;

POWO= Royal Botanic Gardens Kew (2023) Plants of the world Online. http:// <https://powo.science.kew.org/> . Online sources accessed 04 Feburary 2023.

General distribution of other *Larix* spp. from Karlman (2010); *Pinus* spp. from Critchfield and Little (1966):

- Karlman, Lars (2010) Genetic variation in frost tolerance, juvenile growth and timber production in Russian larches (*Larix* Mill.)- implications for use in Sweden. PhD Dissertation, Swedish University of Agricultural Sciences, Umeå. *Acta Universitatis Agriculturae Sueciae* 2010: 30
- Critchfield, William B. and Little, E. L. (1966) *Geographic distribution of the pines of the world*. Washington, D.C, U.S. Dept. of Agriculture, Forest Service

**References for these distributions of Pistacia species (Fig, 7B):**

- Kozhoridze, G., Orlovsky, N., Orlovsky, L., Blumberg, D. G., & Golan‐Goldhirsh, A. (2015). Geographic distribution and migration pathways of Pistacia–present, past and future. *Ecography*, *38*(11), 1141-1154.
- Paludosi, S., Hadj-Hassan, A., eds. (2001) Towards a comprehensive documentation and use of Pistacia genetic diversity in Central and West Asia, North Africa and Europe. Rome: IPGRI
- Dinies, M., Neef, R., Plessen, B., Kürschner, H. (2016). Holocene vegetation, climate, land use and plant cultivation in the Tayma region, northwestern Arabia. In: Luciani, M. (ed). The archaeology of North Arabia: oases and landscapes; proceedings of the international congress held at the University of Vienna, 5-8 December, 2013. Austrian Academy of Sciences Press, Wien, 57–77
- Dinies, M., Plessen, B., Neef, R., Kürschner, H. (2015). When the desert was green: Grassland expansion during the early Holocene in northwestern Arabia. Quaternary International 382:293–302.

**References for dipterocarps core distributions (Fig, 7B):**

- Deb, J. C., Phinn, S., Butt, N., & McAlpine, C. A. (2017). The impact of climate change on the distribution of two threatened Dipterocarp trees. *Ecology and Evolution*, *7*(7), 2238-2248.
- Maury-Lechon, G., Curtet, L. (1998) Biogeography and Evolutionary systematics of Dipterocarpaceae. In: Appanah, S. and Turnbull, J. M. (eds.) A review of Dipterocarps: taxonomy, ecology and silviculture. Bogor, Indonesia: Center for International Forestry Research. Pp. 5-44
- With consultation of data in gbif.org
